# Intraoperative volume restriction in esophageal cancer surgery: an exploratory randomized clinical trial

**DOI:** 10.3325/cmj.2015.56.290

**Published:** 2015-06

**Authors:** Maja Karaman, Ilić, Goran Madžarac, Jana Kogler, Dinko Stančić-Rokotov, Nevenka Hodoba

**Affiliations:** 1 Department of Anesthesiology University Hospital Centre Zagreb, Zagreb, Croatia; 2 Department of Thoracic Surgery “Jordanovac,” University Hospital Centre Zagreb, Zagreb, Croatia

## Abstract

**Aim:**

To investigate whether the fluid volume administered during esophageal cancer surgery affects pulmonary gas exchange and tissue perfusion.

**Methods:**

An exploratory single-center randomized clinical trial was performed. Patients with esophageal cancer who underwent Lewis-Tanner procedure between June 2011 and August 2012 at the Department of Thoracic surgery “Jordanovac”, Zagreb were analyzed. Patients were randomized (1:1) to receive a restrictive volume of intraoperative fluid (≤8 mL/kg/h) or a liberal volume (>8 mL/kg/h). Changes in oxygen partial pressure (Pao_2_), inspired oxygen fraction (FiO_2_), creatinine, and lactate were measured during and after surgery.

**Results:**

Overall 16 patients were randomized and they all were analyzed (restrictive group n = 8, liberal group n = 8). The baseline value Pao_2_/FiO_2_ ratio (restrictive) was 345.01 ± 35.31 and the value six hours after extubation was 315.51 ± 32.91; the baseline Pao_2_/FiO_2_ ratio (liberal) was 330.11 ± 34.71 and the value six hours after extubation was 307.11 ± 30.31. The baseline creatinine value (restrictive) was 91.91 ± 12.67 and the value six hours after extubation was 100.88 ± 18.33; the baseline creatinine value (liberal) was 90.88 ± 14.99 and the value six hours after extubation was 93.51 ± 16.37. The baseline lactate value (restrictive) was 3.93 ± 1.33 and the value six hours after extubation was 2.69 ± 0.91. The baseline lactate value (liberal) was 3.26 ± 1.25 and the value six hours after extubation was 2.40 ± 1.08. The two groups showed no significant differences in Pao_2_/FiO_2_ ratio (*P* = 0.410), creatinine (*P* = 0.410), or lactate (*P* = 0.574).

**Conclusions:**

Restriction of intraoperative applied volume does not significantly affect pulmonary exchange function or tissue perfusion in patients undergoing surgical treatment for esophageal cancer.

Trial registration number: Clinical Trials NCT 02033213.

Pulmonary complications remain a primary cause of morbidity after esophageal cancer surgery. Complications range from atelectasis and pneumonia to acute lung injury and acute respiratory distress syndrome; the risk of these complications is determined largely by preoperative pulmonary status and surgical approach (1). Another factor that can influence the risk of postoperative respiratory complications is the volume of fluid administered intraoperatively (2,3). Such fluid administration is a routine procedure during lung and esophageal surgery (4).

The optimal type and volume of fluid are controversial issues and have not been standardized in international guidelines (5). Several studies suggest that restrictive intraoperative fluid resuscitation during open abdominal surgeries is superior to an aggressive or “liberal” fluid protocol, because it is associated with fewer postoperative complications and shorter discharge time (6-8). On the other hand, restrictive fluid management can lead to hypovolemia and impaired tissue perfusion, which can cause organ dysfunction, particularly postoperative acute kidney injury (9).

In esophageal surgery, fluid management is a special concern because one-lung ventilation (OLV), which is an integral part of anesthesia, can cause postoperative pulmonary edema (10-13). When conventional ventilation is reestablished after surgery, reexpansion of the deflated lung can induce oxidative stress that leads to edema (12-15). In this way, OLV may aggravate the postoperative effects of perioperative pulmonary fluid overload (16). The aim of this exploratory trial was to compare the effects of restrictive and liberal fluid resuscitation protocol on pulmonary gas exchange and tissue perfusion.

## Patients and methods

This randomized controlled single-center open-label trial was approved by the Ethics Committee of the Clinic for Pulmonary Diseases “Jordanovac,” Zagreb, Croatia. All patients gave written informed consent before enrollment.

### Patients

37 patients were scheduled for esophageal cancer surgery at the Department of Thoracic surgery “Jordanovac,” University Hospital Centre Zagreb between June 2011 and August 2012. Patients were prospectively enrolled in the study. Exclusion criteria were age younger than 18 years; severe lung disease, chronic renal insufficiency, or a physical status classification>III on the American Society of Anesthesiologists (ASA) scale (17); or impossibility to perform epidural catheter placement or thoraco-phreno-laparotomy. 16 patients met the criteria. Block randomization of patients, block size of 4, was used to allocate participants into two groups. Allocation concealment was ensured by sequentially numbered, opaque, sealed envelopes.

### Intervention

Esophagectomy in all patients was carried out according to the Lewis-Tanner approach (18). This consisted of an initial laparotomy and gastric tube construction, followed by right thoracotomy to excise the tumor and create an esophagogastric anastomosis. All patients received OLV during the thoracic part of the surgery. All patients were administered 5 mg of diazepam by intramuscular injection 30 min prior to surgery and received preoperative antibiotic and antithrombotic prophylaxis.

All patients underwent the same anesthesia protocol that consisted of a combination of epidural analgesia and general anesthesia. One day before surgery, they received an epidural catheter at level Th4-Th6. General anesthesia was induced by intravenous administration of midazolam (0.07 mg/kg), followed 5 min later by propofol (0.5 mg/kg), fentanyl (2 µg/kg), and rocuronium (0.6 mg/kg). Anesthesia was maintained with inhalation of sevofluran (0.8%) in an oxygen/air mixture. The inspired oxygen fraction (FiO_2_) was titrated to maintain arterial oxygen partial pressure (Pao_2_) above 85 mm Hg. Fentanyl was administered when clinically required, while rocuronium was administered according to the train-of-four ratio. Pressure-controlled ventilation was used and adjusted to achieve a tidal volume of 6-8 mL/kg and an arterial carbon dioxide partial pressure of 35-45 mm Hg. When hemodynamics permitted, a positive end expiratory pressure (PEEP) of 5 cmH_2_O was applied.

A pulmonary artery catheter (PAC) was placed through the right subclavian vein. Data on invasive artery pressure, Pao_2_, and levels of creatinine and lactate were obtained through a cannula inserted in the right radial artery. The functional preload parameters obtained from the PAC, which normally serve as our hemodynamic gold standard, were not used intra-operatively because of the catheter's unreliability when used in the lateral decubital position during open-chest procedures.

During the surgery, one group of patients received ≤8 mL/kg/h of intraoperative fluid (“restrictive group”) and the other received >8 mL/kg/h of intraoperative fluid (“liberal group”). The primary crystalloid used was Plasma-Lyte 148 (pH 7.4; Viaflo, Baxter, Deerfield, IL, US). All patients were administered 10% Aminoven (Fresenius Kabi AG, Bad Homburg, Germany) at 0.5 mL/kg/h. A bolus of 5 mL/kg of colloid (6% Voluven 130/0.4, Fresenius Kabi AG) was administered in order to maintain mean arterial pressure (MAP) above 60 mm Hg. Packed red blood cells were supplied when hematocrit was ≤0.27 L/L. The difference in the amount of intraoperatively administered fluids pertained on the supplementation of crystalloids.

During surgery, a mixture of sufentanyl (50 µg) and 0.5% chirocaine (10 mL) in a total volume of 50 mL of saline was administered at 5-10 mL/h through the epidural catheter. All patients were intubated on the left side with a Robertshaw double lumen tube (Teleflex Medical, Ireland), the position of which was adjusted using a fiber-optic bronchoscope.

Data on Pao_2_, FiO_2_, and the ratio Pao_2_/FiO_2_ were collected 10 min after anesthesia was induced, 30 min after OLV was begun, and 6 h after extubation. The first measurement was considered baseline, while the measurement taken at 6 h after extubation was considered a dependent variable. Baseline measurement for metabolic markers, creatinine and lactate, were performed 10 min after anesthesia induction. Second measurement was performed 6 h after extubation.

### Statistical analysis

Statistical analysis was performed using SPSS (IBM, Armonk, NY, USA), 13.0 software package. Since the Kolmogorov-Smirnov test showed normal distribution data, results are reported as mean ± standard deviation. ANOVA tests were performed to test the differences between the study groups. Independent-sample *t* tests were used to test the differences within the groups for each of the two sets of measurements separately (10 minutes after anesthesia induction and 6 hours after surgery). *P* < 0.05 was considered significant.

## Results

There were no significant differences between the groups in duration of surgery, duration of OLV, number of patients who received noradrenalin intraoperatively, and type and amount of fluids administered intraoperatively (Table 1).

**Table 1:** Characteristics of patients and selected intraoperative data (mean ± standard deviation) on esophageal cancer surgery for patients subjected to restrictive or liberal fluid management

Patients’ characteristics	All patients (n = 16)	Restrictive group (n = 8)	Liberal group (n = 8)
Age, yr	53.12 ± 1.81	53.91 ± 8.31	52.41 ± 13.41
Sex, F/M	6/10	2/6	4/4
American Society of Anesthesiologists classification (17)	2.71 ± 0.48	2.61 ± 0.51	2.75 ± 0.46
Body weight, kg	68.11 ± 17.61	70.01 ± 18.91	66.31 ± 12.61
Duration of surgery, min	300.01 ± 104.21	275.61 ± 91.21	324.41 ± 116.61
Duration of one-lung ventilation, min	158.41 ± 82.91	143.81 ± 68.41	173.11 ± 97.61
Patients receiving noradrenaline, n	9	4	5
Crystalloid administered, mL/kg/h	6.91 ± 2.61	4.81 ± 1.31	9.05 ± 1.81
Colloid administered, mL/kg/h	1.07 ± 0.51	1.12 ± 0.61	1.02 ± 0.37
10% Aminoven, mL/kg/h	0.5	0.5	0.5
10% Aminoven, total, mL	199.06 ± 68.14	212.51 ± 81.61	185.61 ± 53.71
Packed red blood cells, mL	0.44 ± 0.41	0.44 ± 0.41	0.44 ± 0.38
Total volume, mL	3391.88 ± 1022.89	2823.75 ± 965.84	3960.00 ± 755.95
Intraoperative volume, mL/kg/h	8.92 ± 2.64	6.76 ± 1.21	11.08 ± 1.31

In both groups Pao_2_/FiO_2_ ratio was significantly higher at the baseline than 6 h post extubation (restrictive group *t* = 1.46, df = 7, *P* = 0.189; liberal group *t* = 2.03, df = 7, *P* = 0.010). Although there were differences within the groups, there were no differences between the groups (ANOVA complex ANOVA: F_1,14_ = 0.72, *P* = 0.410) (Table 2.)

**Table 2:** Pulmonary gas exchange (Pao_2_/FiO_2_ ratio, mmHg**)** before and after esophageal cancer surgery using restrictive or liberal fluid management

Time point	Restrictive group (mean ± standard deviation)	Liberal group (mean ± standard deviation)	Difference	*P* value*
10 min after anesthesia induction	345.01 ± 35.31	330.11 ± 34.71	14.88	0.410
6 h after surgery	315.51 ± 32.91	307.11 ± 30.31	8.00	0.621

*ANOVA.

In addition to monitoring gas exchange, we also monitored creatinine level as an indicator of renal perfusion and lactate level as an indicator of overall tissue perfusion (Tables 3-4). These levels were measured 10 minutes after anesthesia induction and 6 hours after surgery. There was no significant difference between the intra- and postoperative levels of creatinine either in restrictive (*t* = 0.33, df = 7, *P* = 0.749) or liberal group (*t* = 1.09, df = 7, *P* = 0.310). There was also no significant difference in creatinine levels between the groups at either time point (complex ANOVA: F_1,14_ = 0.72, *P* = 0.410) (Table 3).

**Table 3:** Creatinine (µmol/L) as tissue perfusion indicator during esophageal cancer surgery using restrictive or liberal fluid management

Time point	Restrictive group (mean ± standard deviation)	Liberal group (mean ± standard deviation)	Difference	*P* value*
10 min after anesthesia induction	91.91 ± 12.67	90.88 ± 14.99	1.87	0.791
6 h after surgery	93.51 ± 16.37	100.88 ± 18.33	7.38	0.410

*ANOVA.

**Table 4:** Lactate (mmol/L) as tissue perfusion indicator during esophageal cancer surgery using restrictive or liberal fluid management

Time point	Restrictive group	Liberal group	Difference	*P* value^†^
10 min after anesthesia induction	3.93 ± 1.33	3.26 ± 1.25	0.66	0.322
6 h after surgery	2.69 ± 0.91	2.40 ± 1.08	0.29	0.574
*P* values*	0.030	0.096		

*t test for independent samples.

**†**ANOVA.

Lactate levels behaved slightly different from creatinine levels. The postoperative levels decreased in the liberal group but not significantly (*t* = 1.96, df = 7, *P* = 0.096), while in the restrictive group the decrease was significant (*t* = 2.72, df = 7, *P* = 0.030; Table 4). There was also no significant difference in lactate levels between the groups at either time point (complex ANOVA: F_1,14_ = 0.33, *P* = 0.574; Table 4).

## Discussion

High incidence of postoperative pulmonary edema after esophagectomy (19-22), coupled with reports that OLV can cause pulmonary edema (16,19), led us to examine whether a less aggressive intraoperative fluid approach had an effect on pulmonary gas exchange and tissue perfusion and thus, on the incidence of pulmonary postoperative complications. The planned research time was one year during which time approximately 30-40 esophageal cancer surgeries are performed in our hospital. This fact alone makes us the largest medical center in Croatia that performs esophageal cancer surgery. From 37 patients scheduled for surgery in this period, only 16 patients met the study criteria. Our findings, on this limited sample, suggest that restrictive and liberal fluid management are associated with similar pulmonary gas exchange and tissue perfusion. Therefore, reducing the risk of postoperative pulmonary complications may require some modifications to surgical technique.

We tested two protocols for intraoperative fluid management, restrictive and liberal, with the cut-off defined as 8 mL/kg/h. The amount or type of fluids administered intraoperatively are not standardized. We used the cut-off based primarily based on the experience from our hospital for avoiding intraoperative hypovolemic episodes. The actual rates of fluid administration in our study ranged from 5.0 to 13.6 mL/kg/h, corresponding to 1750-3270 mL administered to the restrictive group and 2500-4840 mL to the liberal group. Previous studies report a range from 4 to 20 mL/kg/h, with total volumes of 1408-2740 mL administered to restrictive groups and 2750-5388 mL to liberal groups (6,23-33).

We focused on the Pao_2_/FiO_2_ ratio as a key indicator of pulmonary gas exchange. Introducing OLV caused the ratio to fall dramatically and reach a minimum by 30 min. Our observation that OLV reduces pulmonary gas exchange is consistent with previous reports (10-15).

Hypoxic pulmonary vasoconstriction in the non-ventilated lung is believed to be the most important variable determining Pao_2_ during one-lung anesthesia (34). Hypoxic pulmonary vasoconstriction is inhibited by a wide variety of physical disturbances and by essentially all volatile anesthetics. During surgery in the lateral position, gravity will usually ameliorate the decrease in oxygenation due to one-lung anesthesia. A third of the shunt during OLV occurs due ventilation perfusion mismatch in the ventilated dependent lung (35). In regard to these facts, and with an aim to accurately interpret the influence of intraoperatively infused volume, measurements obtained during OLV were not compared with the baseline results.

After surgery, the Pao_2_/FiO_2_ ratio in both groups returned to nearly preoperative levels within 6 hours. This finding suggests that fluid restriction does not significantly affect gas exchange and that possible pulmonary fluid overload in the liberal fluid management group does not pose a serious risk.

Though the postoperative Pao_2_/FiO_2_ ratio remained above 300 mm Hg in both groups, the final value was moderately lower than the one measured 10 minutes after induction. Although it was not statistically significant, this decline in Pao_2_/FiO_2_ may reflect a combined deleterious effect of the surgical procedure and OLV on pulmonary function. Still the observed trend did not depend on the fluid management approach.

We measured creatinine levels intraoperatively and after surgery as an indicator of renal perfusion in order to examine whether the fluid management protocol affects the occurrence of renal injury. Restrictive fluid management can produce hypovolemia, which can impair tissue perfusion and lead to organ dysfunction, particularly postoperative acute kidney injury (9). Acute kidney injury, which is associated with 60%-90% mortality (36-38), can be detected as elevated levels of serum creatinine (9). We found no significant differences either between pre- and postoperative values within each group or between the groups. In all cases, creatinine levels were within the reference range. These findings suggest that our restrictive intraoperative fluid approach did not compromise renal function.

We also measured lactate levels as an indicator of overall tissue perfusion. Many studies have confirmed the relationship between tissue hypoxia and lactate generation (39-41). Increases in lactate levels indicate tissue hypoxia due to hypoperfusion. The restrictive and liberal fluid management groups showed similar levels of lactate at both time points, and the levels in the restrictive group decreased significantly from before to after surgery. These findings suggest that restrictive intraoperative fluid administration did not adversely affect tissue perfusion.

A major limitation of this study is the limited number of patients. In Croatia, approximately 50 surgeries of esophageal cancer are performed annually, and 80%-90% are done in our University Hospital Centre. Patients with esophageal cancer are mainly older than 65 with many comorbidities, thus many of them cannot be included in this type of studies. This randomized trial with a small sample of patients suggests that the particular protocol used for intraoperative fluid management does not significantly influence pulmonary gas exchange or tissue perfusion in patients undergoing esophageal cancer surgery. These findings should be further confirmed in randomized trials involving larger numbers of patients as well as patients undergoing other types of open-abdomen surgery.
